# Supplementary material for: Deletion of wfs1 Impairs Oligodendrocyte Precursor Cells Dorsal Distribution and Myelination Through the wfs1-hmgcs1 Axis in Zebrafish
Source: Biology (Basel). 2026 Aug 16;15(16):1402. doi: 10.3390/biology15161402 (PMC13510151; doi:10.3390/biology15161402)
Supplement: Supplementary file 1 [file biology-15-01402-s001.zip › File S1-Transcriptome sequencing data and differentially expressed genes (DEGs) in wfs1-- zebrafish at 5 dpf/2.KEGG/wfs1KOvsWT/DOWN/src/dre04310.html]

dre04310


- dre:497384

- Down regulated genes

497384\_1(-2.05267237809407)

- dre:565241

- Down regulated genes

565241\_2(-6.09874532631292)

- dre:58072

- Down regulated genes

58072(-6.00118378322126)
- dre:30369

- Down regulated genes

30369\_1(-1.70732701220414)

- dre:792699

- Down regulated genes

792699(-1.84389282152821)

- dre:436718

- Down regulated genes

436718\_1(-6.85571323128554)

- dre:58072

- Down regulated genes

58072(-6.00118378322126)
- dre:30369

- Down regulated genes

30369\_1(-1.70732701220414)

- dre:678649

- Down regulated genes

678649(-1.83750740546989)

- dre:323883

- Down regulated genes

323883(-4.8491500403135)

- dre:100006858

- Down regulated genes

100006858\_1(-1.49071226984431)

- dre:30197

- Down regulated genes

30197(-1.07734564021479)

- dre:58072

- Down regulated genes

58072(-6.00118378322126)
- dre:30369

- Down regulated genes

30369\_1(-1.70732701220414)

- dre:402843

- Down regulated genes

402843\_1(-1.44791592833872)
- dre:798564

- Down regulated genes

798564(-2.23020171904595)

- dre:556087

- Down regulated genes

556087\_1(-6.16688081226611)
- dre:791993

- Down regulated genes

791993(-3.69340367298196)
- dre:30127

- Down regulated genes

30127\_1(-1.48241226857528)

- dre:326875

- Down regulated genes

326875\_1(-2.3136095509867)

Close
